# Quantification of Antibiotic Diffusion in Biofilms Using Gold Nanostar Surface‐enhanced Raman Spectroscopy

**DOI:** 10.1002/advs.202510346

**Published:** 2025-10-21

**Authors:** Wafaa Aljuhani, Yingrui Zhang, Matthew P. Wylie, Colin P. McCoy, Steven E. J. Bell

**Affiliations:** ^1^ School of Chemistry and Chemical Engineering Queen's University Belfast Belfast BT9 5AG UK; ^2^ School of Pharmacy Queen's University Belfast Belfast BT9 7BL UK

**Keywords:** antibiotics, biofilm, diffusion coefficient, SERS, Susceptibility

## Abstract

The increased resistance to antibiotics shown by bacteria in biofilms is believed to be partly the result of the limited penetration of antibiotics. However, there are no well‐established techniques which allow quantitative, label‐free monitoring of antibiotic transport in biofilms. Here, it is shown that surface‐enhanced Raman spectroscopy (SERS) with gold nanostars (NS) can be used for the detection of levofloxacin (Levo) in *Staphylococcus aureus* biofilms at clinically relevant concentrations. Ex situ studies showed that although matrix interference reduced the sensitivity compared to aqueous solutions, quantitative detection remained possible. With intact biofilms, monitoring the SERS signals from layers of NS embedded at specific depths allowed the time‐dependence of the penetration of Levo from the surface to the embedded layer to be measured and the diffusion coefficient of Levo to be calculated. The measured value of *D* = 2.79 ± 0.79 × 10^−9^ cm^2^ s^−1^ is over three orders of magnitude lower than in aqueous solutions. This work is the first demonstration that SERS can be a powerful method for investigating antibiotic transport in biofilms, offering new insights into resistance mechanisms and supporting the development of more effective antimicrobial strategies.

## Graphical abstract

Gold nanostar‐enhanced Raman spectroscopy (SERS) enables real‐time, label‐free tracking of levofloxacin diffusion in intact biofilms. Nanostars embedded 16 µm below the surface detect drug arrival over time, revealing the impact of the biofilm matrix on antibiotic transport.

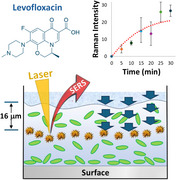

## 1 Introduction

Biofilms are complex microbial communities encased in a self‐produced extracellular matrix (EPS) composed of carbohydrates, proteins, and extracellular DNA. Their inherent antibiotic resistance, which can be 10‐ to 1000‐fold higher than planktonic bacteria, poses significant challenges in both clinical and industrial settings. This resistance is believed to arise primarily from limited antibiotic penetration, where the EPS matrix and cell clusters act as physical barriers while molecular interactions between the drugs and EPS, such as electrostatic binding and hydrophobic interactions, further hinder diffusion and allow bacteria to activate defensive mechanisms before antibiotics reach inhibitory concentrations.^[^ ^1^, ^2^ ^]^ Understanding how antibiotics diffuse within biofilms and the extent of matrix interference is therefore important for evaluating treatment efficacy and developing effective therapeutic strategies.

Drug monitoring in biofilms is challenging. Confocal laser scanning microscopy (CLSM) is the most successful approach to visualize antibiotic distribution in biofilms, enabling detailed 3D mapping of fluorescently labeled antibiotics as they penetrate the biofilm.^[^ ^3^ ^]^ Stimulated Raman spectroscopy (SRS) offers an alternative approach for real‐time monitoring of fluorescently labeled antibiotics penetration in biofilms.^[^ ^4^ ^]^ However, both methods require that labeled antibiotics are available and assume that they behave in the same way as their unmodified analogs.

Here, we present a label‐free approach for the quantitative detection of antibiotics within biofilms using surface‐enhanced Raman spectroscopy (SERS), which is well known to combine high sensitivity with the molecular specificity required for complex biological environments. SERS enhances Raman scattering through the interaction of plasmonic nanoparticles with light and has emerged as a powerful tool for analyzing both synthetic drug molecules and biological samples.^[^ ^5^, ^6^ ^]^ It has already been shown to be capable of detecting antibiotics such as tetracycline, levofloxacin, and ampicillin at low concentrations,^[^ ^6^, ^7^, ^8^, ^9^ ^]^ not only in aqueous solutions but also in complex biological matrices such as milk, urine, and fish tissue.^[^ ^10^, ^11^, ^12^ ^]^ However, the strong affinity of many biological molecules and biopolymers for metal nanoparticles presents a significant challenge for SERS, since these matrix components can give interfering background signals or can reduce the signals from target molecules by blocking access to the surface or hindering aggregation.^[^ ^13^ ^]^ These effects, along with the biocompatibility of enhancing metal nanoparticles and challenges with heterogeneity, are well recognized and have been discussed in excellent reviews.^[^ ^14^, ^15^ ^]^

Biofilms are particularly challenging to study using SERS due to the presence of the gel‐like extracellular polymeric substance (EPS), which means that the samples are viscous, non‐uniform, and contain multiple components that may interact with the enhancing medium.^[^ ^16^ ^]^ As a result, previous SERS studies on biofilms have been limited to detecting biofilm‐associated changes following antibiotic exposure rather than directly detecting antibiotics themselves, and obtaining high‐quality spectra has been challenging.^[^ ^17^, ^18^, ^19^, ^20^, ^21^ ^]^ While some success has been achieved using SERS to monitor biofilm growth through the detection of pyocyanin, a quorum signaling molecule with a strong Raman signature, on solid SERS substrates, achieving quantitative SERS measurements in biofilms remains difficult due to signal variability, competitive adsorption, and unpredictable matrix interferences, all of which reduce reproducibility and sensitivity.^[^ ^22^, ^23^ ^]^ Recent work by Garg et al. introduced engineered nanoplasmonic meshes for spatiotemporal multimodal SERS analysis, enabling diffusion measurements of small molecules in gel‐based systems and in situ biofilm profiling. However, their studies did not examine antibiotic transport within biofilms. In contrast, our approach which embeds gold nanostars directly inside intact biofilms allows label‐free, depth‐resolved monitoring of antibiotic diffusion under more physiologically relevant conditions.^[^ ^24^, ^25^ ^]^

In our previous work, we used SERS to detect molecules in ex situ biofilms where biofilms were diluted and extracted from culture plates to create a controllable homogeneous liquid that retained the chemical composition of intact biofilms. This approach was combined with gold nanostars (NS), which were chosen because they show appropriate plasmonic activity without requiring aggregation, allowing more uniform dispersion of the enhancing particles. This increased uniformity significantly improved the reproducibility of the measurements compared to conventional aggregated spherical or quasi‐spherical particles. In addition, it was found that the use of NS reduced problems of matrix interference caused by the biofilm components, although it did not completely eliminate these effects.^[^ ^26^ ^]^ Building on this work, the current study explores the potential of NS as plasmonic probes for quantitative antibiotic detection and real‐time drug penetration monitoring in biofilms.

In this study, we have found that SERS with NS can be used to quantitatively detect levofloxacin (Levo), a broad‐spectrum antibiotic, in ex situ biofilms. Although measurement sensitivity was significantly lower than was found for simple aqueous solutions, detection at clinically relevant concentrations was still possible without the use of extrinsic labels. Extending this work to intact biofilms, we successfully achieved real‐time, label‐free monitoring of antibiotic diffusion within the biofilm matrix. In this case non‐fluorescent labeling strategies such as isotopic substitution, which have previously been used in Raman‐based diffusion studies, were not necessary because the signals of the target molecule were already readily distinguishable from the background.^[^ ^27^ ^]^

By embedding a thin layer of NS at specific depths within the biofilm and introducing Levo at the surface, we tracked its gradual penetration through signals detected from the embedded NS layer. This allowed the diffusion coefficient of Levo to be quantified, providing insight into how drug molecules interact with and move through the biofilm matrix.

This study directly addresses the challenge of quantitative drug monitoring in biofilms using metal nanoparticle sensors. It represents the first demonstration of SERS for both detecting an antibiotic within biofilms and tracking its penetration in real‐time. It establishes that, with the correct enhancing media, such measurements are possible despite the complexity of the biofilm matrix. Moreover, this label‐free approach lays the groundwork for future investigations into how different antibiotics interact with and penetrate biofilms, contributing to the development of more effective treatment strategies.

## 2 Results and Discussion

Our previous studies with ex situ biofilms showed that the performance of SERS substrates was compromised when they were used in EPS matrices and that this was due to a combination of blocking of surface sites and/or reduced aggregation. It was found that the use of NS that do not require aggregation gave the best results in these challenging media.^[^ ^26^, ^28^ ^]^ Here the first task was to establish if the NS retained sufficient sensitivity in ex situ *Staphylococcus aureus* (*S. aureus*) biofilms to allow detection of antibiotics in a clinically relevant concentration range or was so reduced under these conditions that they could not be used for quantitative measurements.

**Figure 1:**
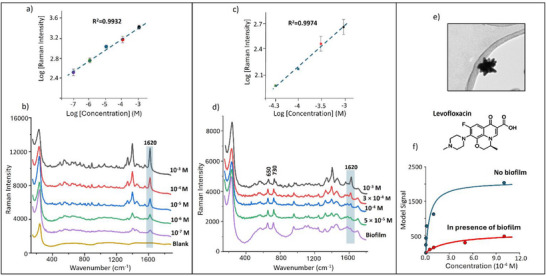
a,c) Log–log plots of SERS peak intensity versus concentration for Levo, in the absence and presence of ex situ S. aureus biofilm, respectively. b,d) SERS spectra of Levo at the marked concentrations in the absence and presence of ex situ biofilm, respectively. All spectra are the average of three technical replicates recorded on the same day using the same colloid batch. e) TEM image of the NS. f) Langmuir‐type plots of the SERS intensity data are shown in (a,c). The solid lines shown are fits to the Langmuir isotherm.

Levofloxacin (Levo), a broad‐spectrum fluoroquinolone, was chosen for this study due to its distinct SERS bands at 1620 and 1395 cm^−1^, which are associated with the C═C vibration of the quinolone ring system and a combination of the quinoline ring with symmetric (COO^−^) stretching.^[^ ^7^ ^]^ These characteristic signals are clearly observed in the SERS signals of Levo in the absence of *S. aureus* biofilm, as shown in **Figure** **1**b. However, for all the measurements in ex situ biofilms, the peak at 1620 cm^−1^ was chosen to monitor the SERS signals of Levo since the 1395 cm^−1^ band overlaps with features in the biofilm spectrum (Figure 1d).

Figure 1b shows a series of SERS spectra of Levo in aqueous solution over the concentration range 10^−7^–10^−3^ mol dm^−3^. The spectra increase in intensity with concentration, while clear signals much larger than 3x the noise level are observed at 10^−7^ mol dm^−3^, meaning that the limit of detection in a simple aqueous solution is significantly below 10^−7^ mol dm^−3^.

The Levo detection experiments were then repeated using the same conditions, apart from the addition of ex situ biofilm. Figure 1d shows the resulting spectra. The spectrum of the biofilm alone shows numerous distinct peaks, the most obvious being those at 650 and 728 cm^−1^ which are associated with nucleic acids.^[^ ^29^ ^]^ Addition of Levo at high concentration (10^−3 ^mol dm^−3^) gave spectra where the strong characteristic bands at 1395 and 1620 cm^−1^ grew to be stronger than those of the biofilm matrix, although they were lower intensity than the equivalent bands in aqueous solution at the same concentration. However, the intensity of the signals dropped off rapidly with reduced concentration, resulting in a limit of detection ≈5 × 10^−5^ mol dm^−3^, which is significantly higher than that of the aqueous measurements. To give a context for these values, for Levo the MIC (minimum inhibitory concentration) for *Staphylococcus aureus* colonies grown on Ti coupons is typically reported as 1 µg mL^−1^ (4.89 × 10^−6^ mol dm^−3^), which is within the concentration range of the SERS measurements in simple aqueous solution but outside the compromised range found with ex situ biofilm.^[^ ^30^ ^]^ However, under more clinically relevant conditions, which are also comparable to this study (*S. aureus* strain ATCC 6538 in biofilm with starting inocula 10^9^ cfu mL^−1^) the minimum bactericidal concentration (MBC) was found to be 9.6 mg mL^−1^ (2.7 × 10^−2^ mol dm^−3^), i.e., more than three orders of magnitude larger than in simple culture and within the range which can be achieved in ex situ biofilm.^[^ ^30^ ^]^

Since the calibration data span several orders of concentration, they are shown as log–log plots in Figure 1a,c. The data show approximately linear behavior, as is common in quantitative SERS measurements. These plots also illustrate the reproducibility of the measurements with error bars showing ± 1 relative standard deviation (RSD) obtained from three repeat measurements. Although these log–log plots are useful for measuring concentrations, they are based on the Freundlich isotherm, which is an empirical equation, so the parameters have no simple physical interpretation, although in general terms they can be regarded as reflecting the presence of surface sites with different binding properties. For this reason, the data were also fitted to the standard Langmuir isotherm, which in this case would have the form:

(1) $$\frac{S_{c}}{S_{\max}}=\frac{K\left[\mathrm{Levo}\right]}{1+K\left[\mathrm{Levo}\right]}$$

Regression plots of concentration versus concentration/signal allowed values for *S_max_* and *K* to be estimated. The isotherms obtained using these values are superimposed on the experimental data in the plots shown in Figure 1f. These values can be used to give a semi‐quantitative description of the binding, which shows that the *K* value is dramatically decreased from 20 000 to 3000 in the presence of the biofilm and is consistent with access to the surface being hindered by the EPS. In addition, the 3x reduction in *S_max_* from 2075 to 588 suggests that, in addition to increased hinderance to active surface sites, the total number of sites is also reduced to 1/3 of the value in aqueous solution, presumably because these sites are completely blocked. This interpretation gives an approximate picture of the analyte adsorption but should be treated with caution, partly because of the experimental uncertainty associated with fitting the regression but principally because the experimental data do not fit the Langmuir model well. This should not be surprising since the Langmuir model assumes the binding sites are homogeneous, while even the interpretation above requires two different populations of surface sites, those which are accessible but hindered and those which are completely blocked. In reality there are most likely a range of surface sites which are affected to different extents by the presence of the biofilm and indeed this is consistent with the fact that the data fit a Freundlich isotherm, as shown in Figure 1a,c.

While the experiments with the ex situ biofilm are important in establishing that the matrix does not prevent quantitative measurements of the antibiotic concentration in the appropriate analytical range, they do not allow for any detailed understanding of the factors that lead to the significant difference in sensitivity which is observed between the biofilm and simple aqueous samples. Attributing the lower signal intensity of Levo in the biofilm matrix is challenging considering the numerous different factors at play in these type of measurements. One possible explanation is the competition between Levo and the matrix components for binding to the metallic surface, which is a common problem when using SERS for direct analyte detection of target analytes in complex biological environments. In particular, biological environments are rich in adenine, proteins, and polysaccharides that can adsorb to the surface of unmodified nanoparticles like the NS colloid used here, competing with the antibiotic molecules or hindering their access to the enhancing surface.^[^ ^14^ ^]^ This interpretation is consistent with our previous findings in ex situ biofilm systems, where signal suppression was shown to arise from coupled equilibria involving multiple matrix components, rather than interference by a single dominant species.^[^ ^28^ ^]^

**Figure 2:**
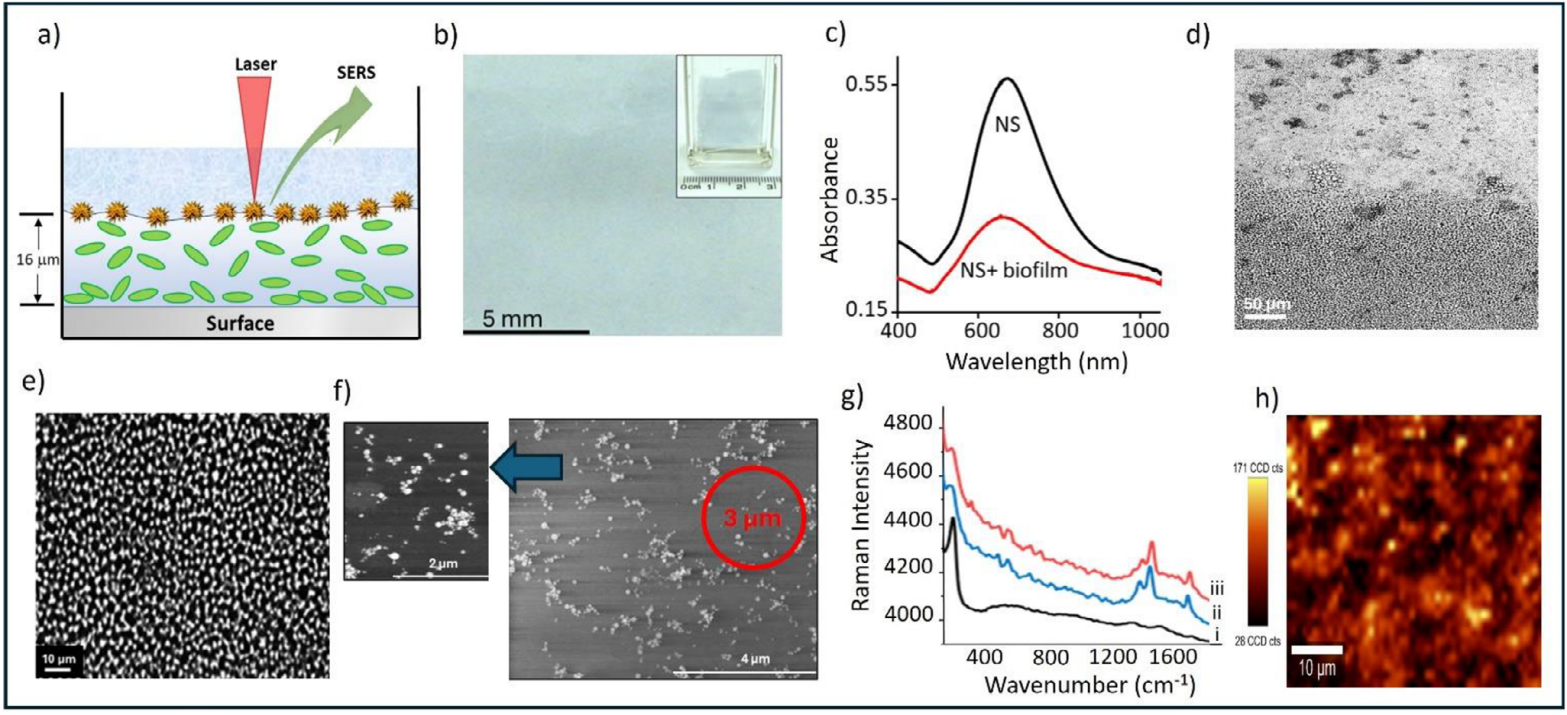
a) Schematic representation of the experiment, showing the incorporation of NS on the biofilm surface. b) Photograph of the surface‐bound NS on a biofilm grown on a quartz slide. The insert shows the entire slide including the area of biofilm which was not immersed in the NS solution, which is colorless and transparent c) Extinction spectrum of the surface‐bound NS compared to the NS colloid. d) Optical image of a biofilm which had been partly submerged in an NS solution, highlighting the contrast between the untreated biofilm region (top) and the immersed region which carries a layer of surface‐bound NS (bottom) which are uniformly distributed at a length scale of > 300 µm. e) Higher‐magnification image of the surface‐bound NS, revealing finer structural details of the NS layer at a length scale of <10 µm. f) SEM images of the surface‐bound NS, showing NS distributed across the biofilm surface rather than embedded within the EPS matrix (scale bar = 4 µm). The inset image provides a higher magnification view. The 3 µm laser spot diameter is indicated by the red circle, illustrating that the laser spot size is slightly too small to average over the non‐uniform particle distribution. g) SERS spectra of the surface‐bound NS prior to Levo addition i) as well as 2 min ii) and 8 min iii) after Levo addition. h) SERS mapping of the chloride signal (233 cm^−1^) of the surface‐bound NS, scale bar = 10 µm.

While ex situ experiments are useful in allowing the average interactions of biofilm components with the surface to be probed, the sample preparation removes all the information about the spatial distribution of these components within intact biofilms. To measure this, the particles must be located within the biofilm itself. The most obvious approach to addressing this problem would be to incubate the biofilm samples with a colloidal suspension of the nanoparticles, and with small (< 25 nm) NPs, it is known that the NPs can penetrate into the biofilm. Indeed, this is being investigated as a method of drug delivery into biofilms.^[^ ^31^ ^]^ However, in the current study the NPs are > 100 nm NS, which would be expected to show low penetration into biofilms. In the current experiments, incubation of a blue‐colored 5 × 10^10^ particles mL^−1^ suspension of NS for 30 min to allow binding, followed by rinsing to remove unbound particles resulted in the biofilm changing from colorless to pale blue, demonstrating that the particles adsorb onto or within the biofilm over this timescale (**Figure** **2**a). Standard optical imaging of these revealed a surprisingly homogeneous distribution of NS across the biofilm surface, evident from the relatively uniform blue layer coating the biofilm (Figure 2b).

Figure 2c compares the extinction spectrum of the particle‐treated biofilm (surface‐bound NS) with that of the NS colloid, demonstrating that the plasmonic properties of the NS remain largely unaffected following incorporation into the biofilm. Notably, the extinction peak at 760 nm remains unshifted, despite a reduction in intensity due to the lower particle concentration, indicating that the local environment around the nanoparticles does not significantly alter their plasmonic properties.

Confocal laser scanning microscopy, which allowed z‐scanning of the intact structures, was used to investigate the extent of NS penetration into the biofilms. In this case, a water immersion objective was used to observe the NS within the intact biofilms. Although it would be expected that optical microscopy would be unsuitable for observing individual NS, it was found that particles used in this study exhibit strong plasmonic light scattering and potentially weak luminescence under 405 nm laser excitation. This allowed them to be detected using HyD detectors, which collected emitted or scattered light in the 557–735 nm range. Using this approach, it was possible to observe a very obvious layer of particles sitting on the surface of the biofilm, which is even more apparent when comparing the treated and untreated regions of the biofilm, as shown in Figure 2d. Since the particles readily allowed the top surface of the biofilm to be located, the thickness of the biofilm could be easily measured by locating the bottom of the biofilm and the top surface of the slide on which it was grown. The data in Figure 2 shows a biofilm that was grown for 4 days which was ≈16 ± 4 µm thick. Optical microscopy also shows that the film is continuous and hole‐free on the length scale of > 300 µm (Figure 2d). At higher magnification, the optical images appear to show that the NS layer has structure on a length scale of < 10 µm (Figure 2e). More accurate mapping of the distribution of the NS was carried out using SEM. Although this meant that dried samples were studied, the images show NS sitting on top of the surface, rather than being located within a matrix of dried EPS. In addition, while the particles show some clustering into small aggregates, which might be the result of the drying process, the particles are distributed over the whole surface rather than being found as large aggregates (Figure 2f).

This particle distribution suggests that it should be possible to detect SERS signals over the entire surface of the biofilm. Figure 2g shows the SERS signal of the particle‐treated biofilm, which is dominated by the same chloride signal at 233 cm^−1^ that is also found in the as‐prepared NS colloid. Figure 2h shows the result of SERS mapping of a typical region of the sample using the chloride signal at 233 cm^−1^, as the SERS marker band. The map shows the local variation in intensity over scales < 10 µm, which is consistent with the particle distribution shown in the SEM image. In this experiment the heterogeneity can be mapped because the laser spot diameter is ≈3 µm. As shown in Figure 2f, which has a circle of 3 µm added for convenience to the reader, this spot is slightly too small to average over the non‐uniform distribution of particles, so at some points it will randomly encounter regions with high or low numbers of enhancing NS. The SEM image also allows us to estimate the particle density, which was found to be ≈5 × 10⁸ particles cm^−^^2^, based on counting the number of particles in a 100 µm^2^ area. It is notable that the strong adenine bands found when NS were mixed with ex situ biofilms are not observed in the intact biofilm SERS signal shown in Figure 2g. This could be due to differences in the concentration of adenine‐containing species between the two types of samples or because the adenine is not uniformly distributed across the surface of the biofilm, so that it is present but is not being detected at the point probed. Attempts to detect locally high concentrations of adenine within mapping data or by sampling individual points over the entire surface did reveal some points (2 out of 21) where adenine signals could be detected but even in these spectra the signal was similar in size or smaller than those found in the ex situ samples (Figure S1, Supporting Information). This suggests that the reason for the differences in adenine signal height between ex situ and intact biofilms is due to a higher adenine concentration within the matrix of the ex situ samples. This is reasonable, since any damage to the constituent bacteria during preparation of the ex situ samples could lead to the release of intracellular components which are rich in adenine‐containing compounds.

The next step was to test if Levo could be detected in intact biofilms. Figure 2g shows the result of adding Levo (10^−3^ mol dm^−3^) to a 4 day biofilm with an adsorbed NS surface layer. The spectra show growth of the Levo signal occurs rapidly, within the 2 min time resolution of the experiment. The signal does not grow beyond this time, as shown by the 8 min spectrum which is also included in Figure 2g. The absolute size of the Levo signals was lower than that obtained with the ex situ samples (Figure 1d) but those had a higher number of enhancing particles in the probed volume. The relative size of the Levo signal compared to that of the chloride blank signal at 2 min was found to be 0.22 which was found to correlate to a concentration of 10^−3^ mol dm^−3^ using the calibration plot of Levo:chloride relative intensity versus Levo concentration established for the ex situ biofilms (Figure S2, Supporting Information). Although this calibration was obtained from homogenized ex situ biofilm samples, it offers a reasonable estimate of the surface concentration in the intact biofilm since in each case the composition of the matrix, which is what determines the interactions with the nanoparticle surface, is the same. The effect of differences in the structural heterogeneity of the ex situ and intact biofilms would be expected to be much less significant. This result is entirely consistent with the experimental conditions where the biofilm was in contact with 10^−3^ mol dm^−3^ solution of Levo and suggests that the Levo concentration in the surface layer of the intact biofilm rapidly becomes equal to that of the Levo solution which it is in contact with.

Since applying NS solely to the biofilm surface does not permit monitoring of Levo penetration within the biofilm, the next step was to incorporate the NS within the biofilm matrix. The fact that the particles did not penetrate the biofilm might be regarded as a disadvantage since it means that it was not possible to simply place the biofilm in a solution of the particles and allow them to diffuse throughout the sample. However, it could be used to advantage since it meant that if the particles were placed at a particular location in the film they remained in place over time. Here, a layer of particles was created within the biofilm at a fixed depth by first growing the biofilm to a desired thickness and then treating it with an NS particle suspension, creating a surface layer, as described above. Since the adsorption and excess particle rinsing process could be carried out quickly (30 min), when the samples were placed back into the growth medium the biofilm continued to grow on top of the added particle layer and after a sufficient time this resulted in a sample where the NS were trapped as thin layer within a thicker living biofilm matrix (Figure S3, Supporting Information). This process obviously allows the depth of the particle layer and overall biofilm thickness to be altered by changing the time the biofilm is allowed to grow before the NS layer is added and the time after particle addition that the biofilm growth is allowed to continue.

**Figure 3:**
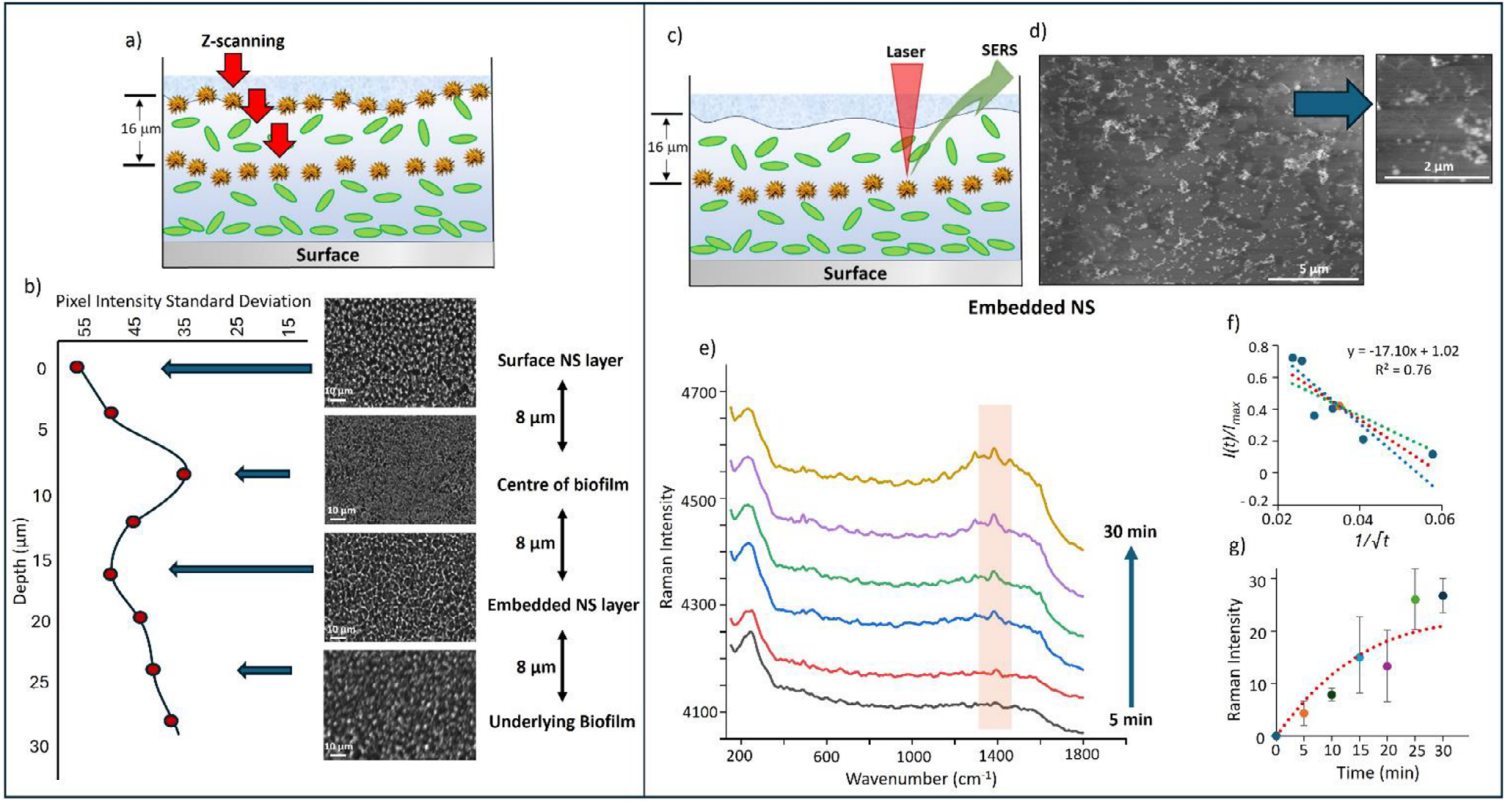
a) Schematic representation of the sandwich biofilm structure, illustrating NS layers at the surface and at a fixed depth within the biofilm matrix. b) Optical widefield microscope images of the biofilm sample with two NS layers. Images show the surface layer and the sample at depths of 8, 16, and 24 µm. Quantification of image contrast using the standard deviation of pixel intensities at different depths in the biofilm is also presented. c) Schematic representation of the NS layer embedded within the biofilm and surrounded by the EPS matrix. d) SEM image of the embedded NS layer, with the inset showing a higher magnification view of the NS within the EPS. e) SERS spectra of Levo recorded at 5 min intervals, highlighting the increase in signal intensity over time as Levo penetrates and accumulates at the NS embedded layer. The spectra are averaged from three independent biofilm samples. f) Linearized plot of I(t)/I_max_ against 1/√t fitted to a simplified model of Fick's second law. The slope of the fitted line was used to calculate the diffusion coefficient (D). g) Plot of the average SERS intensity of the Levo signal at 1396 cm^−1^ as a function of time, with error bars indicating the variation across samples.

The structure of the samples with embedded NS layers can be monitored using similar approaches to those used for the surface layers described above. The main challenges are to confirm that the particles, which form a layer on the surface of the biofilm (as shown by earlier experiments), remain arranged in this way during the subsequent biofilm growth stage and to then measure the depth of this particle layer below the biofilm surface. In this case, optical widefield microscopy with a water immersion objective was used as described above, and it was found that the overlying biofilm did not distort the images significantly, so that optical images of the embedded particle layer within the intact biofilm were very similar to those of the surface layer. Since the biofilms were unstained, the top surface of the biofilms with embedded NS layers was difficult to detect, making it impossible to measure how deep they were below the surface. However, by taking advantage of the fact that the particles adsorb to the outer surface of the biofilm, it was possible to use the NS suspension as a form of localized stain by immersing the sample with an embedded layer back into a NS solution, creating a double sandwich structure with visible particle layers both at the surface and at a fixed depth within the film (**Figure** **3**a). Z‐scan images of the sandwich sample were then captured at 2 µm increments. Images obtained at the surface and at depths of 8, 16, and 24 µm (Figure 3b) show the NS layers are present on the surface and at a depth of 16 µm, while these features are absent in the blank images recorded at 8 and 24 µm. To quantify the extent to which the high contrast pattern characteristic of the NS layers could be observed at each depth in the biofilm, the standard deviation of the pixel intensities of each of the images was measured using Image J software. The resulting data very clearly reflect the layered structure of the biofilm since, as shown in Figure 3b, the values show a high standard deviation at the surface, which decreases on moving deeper into the biofilm but then increases as the image plane approaches the embedded NS layer, maximizing at a depth 16 µm. The values fall again at greater depth as the image plane moves below the embedded particle layer.

Importantly, the loss of the patterned structure in images obtained below the surface shows that the non‐uniformity on the < 10 µm length scale observed on the *x*‐*y* plane (Figure 2e) does not extend deep into the biofilm. The pattern that can be seen to be at the top surface presumably arises because the biofilm has an irregular surface with bumps a few µm high, so that the scattered light from the surface shows bright and dark areas depending on the angle which the particle layer makes with the excitation source. Below this surface layer there is “bulk” biofilm which the surface structure does not reach down to, as demonstrated by the lack of particle penetration (Figure 3b).

Further confirmation that the layered structure contained embedded NS was provided by scanning electron microscopy (SEM), which allowed the appearance of NS on the biofilm surface to be compared with those within the film. On the surface, the NS appear sharp at both low and high magnification, clearly indicating their location on the surface as shown previously in Figure 2f. In contrast, NS embedded within the biofilm's interior are surrounded by extracellular polymeric substances (EPS). The EPS is visible at lower magnification as a layer of dried material with some gaps, which are presumably formed during the drying process but are useful in that they give some contrast which allows the featureless grey polymer to be observed. At higher magnification the embedded NS appear blurred in the SEM images, which further confirms they are located within the dried EPS layer (Figure 3d).

The fact that the NS can be located at a fixed depth within the biofilm enables experiments that measure penetration of drug molecules into the biofilm to be measured in a very straightforward and intuitively attractive way. In this approach a solution of the drug at the required concentration is placed in contact with the surface of the biofilm, which has an embedded NS layer (but no additional surface layer). The SERS signals from the NS layer are monitored over time, and the rise of the drug signal is used to indicate the increase in concentration of the drug at a known depth as it penetrates the biofilm. The 785 nm laser excitation used in this study gives low autofluorescence and effective light penetration. This wavelength has enabled Raman signal detection at depths of ≈1–3 mm in biological matrices, making the 16 µm probe depth used in this study well within the accessible range.^[^ ^32^ ^]^

In the current study, Levo penetration was monitored by recording SERS signals acquired at 5 min intervals. These show the growth of peaks due to Levo over a period of 30 min, indicating gradual accumulation of Levo at the NS layer within the biofilm (Figure 3e). Also shown in the Figure 3g is a plot of the intensity of the Levo SERS signal at 1396 cm^−1^ as a function of time. The data in the Figure are averages of repeat experiments run on three different biofilm samples, each of which was prepared in the same way to give NS layers embedded at 16 µm. The error bars show a variation of just 5% to 23%, which demonstrates that the approach gives good levels of reproducibility considering the challenges of working with biofilms, which include variability in biofilm structure, the heterogeneity of the extracellular matrix, and the complex interactions between biofilm components and nanoparticles.

The increase in signal intensity with time can be used to directly calculate the diffusion coefficient, *D*, for Levo diffusion through the biofilm, using Fick's second law. The form of this equation, which is appropriate for the diffusion of a small molecule penetrating a semi‐infinite medium is:

(2) $$I\left(t\right)=I_{\max}\left(1-\mathrm{erf}\left(\frac{x}{2\sqrt{\mathrm{Dt}}}\right)\right)$$

where *I*(*t*) is the signal intensity at any time *t* after the molecule is introduced at the surface (*x* = 0), *I_max_* is the maximum signal intensity, and *x* is the depth at which the signal is measured.^[^ ^33^ ^]^

The accurate solution of Equation (2) requires the error function corresponding to $\frac{x}{2\sqrt{Dt}}$ to be calculated but the expression can be simplified by retaining only the first term of a Taylor expansion of the error function, which gives:

(3) $$I\left(t\right)=I_{\max}\left(1-\frac{x}{\sqrt{\pi \mathrm{Dt}}}\right)$$

In this study the data were fitted to Equation (3) by plotting *I(t)*/*I_max_* against 1/√*t*. Since intensities did not plateau, *I_max_* was treated as a variable, and in the fitting process the value of *I_max_* was adjusted to give an intercept of 1, and the slope was then used to calculate *D*.

A plot of this type, using the averages of the intensities from three independent experiments, is shown in Figure 3f, along with error bars showing ± 1 standard error in the slope. The *D* and *I_max_* values obtained from Figure 3f were also used to calculate the model line superimposed on the raw data shown in Figure 3g. In this case the accurate Equation (2) was used to calculate intensity values since this allows the value at zero time to be included.

This process provides a straightforward approach to estimating antibiotic diffusion through biofilms and here the averaged data gave a diffusion coefficient for Levo of 2.79 ± 0.79 10^−9^ cm^2^ s^−1^. The 28% uncertainty which arises from the scatter in the intensities is significantly larger than the error introduced by using the Taylor expansion which we estimate as ≈10%. The extent to which sample‐to‐sample variation contributes to the uncertainty can be judged by calculating the diffusion coefficients of the three independently prepared biofilms whose data was averaged above. This gave values of 2.79 ± 0.66, 2.76 ± 1.24, and 2.82 ± 1.26 × 10^−9^ cm^2^ s^−1^, which are closer to each other than would be expected on the basis of the error in each measurement. Presumably this similarity is merely the result of coincidence, but it does suggest that, despite the inherent biochemical and structural heterogeneity of biofilms, differences between the samples are not the main cause of the uncertainty in the averaged data. In addition, it shows that other variables, such as the uncertainty in the depth of the particle layers for example, are not a significant problem, even though *D* scales as *x* ^2^. However, irrespective of the sources of uncertainty, the main conclusion is that the level of precision the method can achieve is more than sufficient to establish the properties of this system, where the important observations are around orders of magnitude changes in *D*.

The most striking result from this study is that the diffusion coefficient of Levo measured in the biofilm is more than three orders of magnitude smaller than the typical diffusion coefficients of antibiotics with molecular weights ranging from 300 to 1400 g mol^−1^ in aqueous solution, which have previously been reported as ≈6 × 10^−6^ cm^2^ s^−1^.^[^ ^34^ ^]^ One possible explanation for this reduction in *D* could be that the biopolymers create a physical barrier to free diffusion of the Levo, which would be expected to be a combination of increased viscosity and tortuosity and is a well‐known physical phenomenon. However, when the diffusion coefficients for antibiotics in the 300 to 1400 g mol^−1^ size range, such as ciprofloxacin, gentamicin and vancomycin were measured in a range of polymer systems mimicking biofilm matrices, such as 2% alginate, 10% porcine gastric mucus, 2% agar, or mixtures of these polymers, it was found that the diffusion coefficients were typically reduced by a factor of ≈1 to 3 compared to their values in water, despite significant differences in molecular weight among the antibiotics tested and variations in the type of polymer used.^[^ ^34^ ^]^ This observation suggests that while molecular weight and polymeric barriers contribute to reduced diffusion in biofilms, this is not sufficient to account for the dramatic reduction of the diffusion coefficient of Levo observed in the current study.

An additional possibility is that interactions between the biofilm components and antibiotics may reduce the rate of diffusion. This has been treated theoretically by Fan et al., who modeled diffusion through polymers and contrasted the situation where there were no interactions to the cases where the antibiotics either reversibly adsorbed to the polymer matrix or were irreversibly bound.^[^ ^35^ ^]^ These models showed that adsorption to the polymer could dramatically reduce diffusion rates. Since biofilms are intricate matrices composed of polysaccharides, proteins, nucleic acids, and other molecules there are numerous possibilities for a given antibiotic to interact with at least one of these components and therefore reduce diffusion compared to the non‐interacting case.^[^ ^36^ ^]^ Some examples of this effect are known, for example, significant penetration of ciprofloxacin into a *P. aeruginosa* biofilm ≈233 µm thick was found to occur within 4 h while, in contrast, tobramycin, a cationic molecule that is believed to bind strongly to polysaccharides within the biofilm, required more than 36 h to reach a comparable level of penetration.^[^ ^37^, ^38^ ^]^ This observation shows that binding interactions can dramatically alter the rate of antibiotic diffusion in biofilms, independent of any physical factors.

In addition to polysaccharides, Levo is known to bind strongly to other components in the biofilm listed above. For example, its interaction with ds‐DNA is sufficiently strong that it has formed the basis for electrochemical Levo sensors, so binding between the Levo and e‐DNA in the biofilms might be expected.^[^ ^39^ ^]^ Similarly, significant adsorption of Levo onto human plasma proteins and bovine serum albumin has also been observed, suggesting that Levo‐protein interactions might also be significant in the biofilm.^[^ ^40^, ^41^ ^]^ It is difficult to determine which of these interactions might be dominant, particularly since the interaction between Levo and even a single component can itself be complex. For example, docking analysis of the interaction of Levo with albumin showed it involved a combination of electrostatic, hydrogen bonding, and hydrophobic interactions,^[^ ^41^ ^]^ while molecular modeling showed van der Waals/hydrogen bond forces are responsible for minor grove binding of Levo to DNA.^[^ ^42^ ^]^

The low *D* found for Levo here is similar to that which was found previously for vancomycin in *S. aureus* biofilms (6 × 10^−10^ cm^2^ s^−1^), which was determined using alkyne labeled vancomycin and simulated Raman spectroscopy (SRS).^[^ ^4^ ^]^ Vancomycin has been shown to covalently bind to polysaccharides, particularly dextran, through its primary amino group which could significantly hinder its diffusion.^[^ ^43^ ^]^ The similarity in *D* values suggests that both Levo and vancomycin have strong interactions with components of biofilm, while the observation that the value of *D* is higher for Levo than vancomycin suggests that its interactions with the matrix may be somewhat weaker. This is consistent with reports that vancomycin exhibits lower penetration in biofilms than Levo (or the closely‐related ciprofloxacin), although specific diffusion coefficient values or detailed explanations for this difference are rarely provided.^[^ ^44^, ^45^ ^]^ However, the main conclusion from the current work is still that although it is difficult to separate out which of the several possible interactions between Levo and components of the matrix is the most important, the net effect is that these interactions are strong enough to dramatically reduce diffusion in the biofilm.

## 3 Conclusion

Detecting antibiotics within biofilms presents a significant challenge due to the complexity of the biofilm matrix, which can interfere with detection methods. This study demonstrated, for the first time, the application of SERS for the label‐free detection of an antibiotic, Levo, in biofilms at clinically relevant concentrations, although with reduced sensitivity compared to measurements in aqueous solutions.

In addition to establishing the potential of SERS for simply detecting antibiotics within biofilms, this study shows that SERS can be used to measure antibiotic penetration with sufficient confidence to give useful results. Embedding NS at specific depths within the biofilm allowed Levo penetration to be monitored and its diffusion coefficient to be measured (*D* = 2.79 ± 0.79 10^−9^ cm^2^ s^−1^). The dramatically reduced diffusion coefficient that Levo displays in the biofilm underscores the significant impact of the biofilm matrix on antibiotic transport. The fact that similar values were measured across three independent samples demonstrates that the method of preparing the biofilms and making the measurements allows reproducible results to be obtained and that anticipated issues with sample‐to‐sample variation and within‐sample heterogeneity, although clearly present, are not so large that they prevent measurement of *D*, even under these challenging conditions.

Overall, this approach provides a robust method for investigating antibiotic diffusion in biofilms, offering insights into matrix‐related resistance and molecular transport. Since it is not confined to Levo and *S. aureus* but is broadly applicable to other antibiotics and biofilm species. In the future it could allow the diffusion of antibiotics with different molecular properties to be compared and underpin the investigation of the effect of factors such as pH, nutrient levels or biofilm maturity on matrix permeability. Similarly, it could be used to evaluate novel therapeutic approaches such as the use of penetration‐enhancing agents or novel delivery systems. Although the current approach measures diffusion at a fixed x,y point in the biofilm sample, future studies could include SERS spatial mapping techniques to investigate heterogeneity within the biofilm matrix, revealing local differences in density or transport dynamics. These capabilities mean that the in situ SERS approach shown here can provide a powerful and versatile new tool which can be used to provide insights into antibiotic resistance mechanisms and support the development more effective strategies for targeting pathogenic bacteria in biofilms.

## Conflict of Interest

The authors declare no conflict of interest.

## Supporting information



Supporting Information

## Data Availability

The data that support the findings of this study are available from the corresponding author upon reasonable request.
