# Supplementary material for: Quantification of Antibiotic Diffusion in Biofilms Using Gold Nanostar Surface‐enhanced Raman Spectroscopy
Source: Adv Sci (Weinh). 2025 Oct 21;13(2):e10346. doi: 10.1002/advs.202510346 (PMC12786373; doi:10.1002/advs.202510346)
Supplement: Supplementary file 1 — Supporting Information [file ADVS-13-e10346-s001.docx]

**1. Methods**

**1.1 Nanostar colloid preparation**

Nanostar colloids (NS) were prepared using a previously reported method.^46^ For *ex-situ* measurements, the colloid was used at its original concentration (10⁹ particles/mL). However, for *in-situ* measurements, the colloid was concentrated by centrifugation to achieve a final concentration of 5×10¹⁰ particles/mL.

**1.2 *Ex-situ* biofilm preparation**

*Ex-situ* biofilms were prepared following an established protocol.^47^ Briefly, an overnight cell culture of *Staphylococcus aureus* (*S. aureus*, ATCC 29213) was diluted in Tryptic Soy Broth (TSB) to obtain a 10^6^ cfu mL^−1^ inoculum. Then, 150 μL of this inoculum was added to each well of a sterilized 96-well microplate (Thermo Scientific) and incubated at 37 °C and 100 rpm for 24 h. The medium was then replaced with 150 μL of fresh TSB and incubated for an additional 24 h. At 48 h, the medium was removed and each well was rinsed three times with water to remove residual medium and unattached cells. 5 μL of sterile water was then added to each well and the plate was sonicated for 15 min to transfer the biofilm attached to the well walls into the water. Finally, the resulting *ex-situ* biofilm solutions were aspirated into a container for SERS analysis.

**1.3.** ***In-situ* biofilm preparation**

For *in-situ* intact biofilm preparation, quartz slides (22 × 22 mm) were incubated with 15 mL of a 10⁶ CFU mL⁻¹ *S. aureus* inoculum and cultured under the same conditions as the *ex-situ* biofilms. After biofilm growth was complete, the slides were gently rinsed with sterile water to remove unattached cells and then were used for analysis.

**1.3.1 Surface-bound NS preparation**

For preparation of the surface-bound NS, 350 μL of the concentrated NS colloid was added to a 4-day old intact biofilm. The colloid was incubated for approximately 30 minutes to allow NS binding to the biofilm but was not allowed to dry. Then, the biofilm was gently washed to remove unbound NS, leaving only surface-bound NS for analysis.

**1.3.2 Embedded NS layer preparation**

For preparation of the embedded NS layer, 350 μL of the concentrated NS colloid was added to the 4-day old biofilm and incubated for 30 minutes, followed by gentle washing to remove unbound NS. The biofilm containing the NS was then returned to the growth medium and allowed to grow for an additional 4 days, during which a layer of EPS formed over the NS, embedding the nanoparticles within the biofilm matrix. Finally, the biofilm was washed gently with water and prepared for analysis.

**1.3.3** **Double-Layer NS preparation**

For biofilms containing two NS layers, a second 30-minute application of 350 μL of concentrated NS colloid was performed on biofilms with an embedded NS layer, resulting in the formation of a second NS layer on the biofilm surface. After gentle washing to remove unbound NS, the biofilm was prepared for analysis.

**1.4 Characterization**

Optical images of biofilms were obtained using widefield fluorescence microscopy with a 25x water immersion objective. Bright-field excitation was performed at 405 nm with detection over the wavelength range of 557–735 nm. Biofilms containing the embedded NS layer were approximately 30 μm thick, while those with only a single NS layer on the surface were approximately 16 μm thick. Z-slices were acquired at 2 μm intervals.

For SEM imaging, a Quanta FEG 250 scanning electron microscope was used to visualize nanoparticles within the intact biofilm.

 For TEM characterization, NS were imaged by Joel JEM-1400 plus Transmission Electron Microscope and TALOS F200X G2: Scanning/transmission electron microscope (S/TEM). Samples were prepared for imaging by adding one drop of the colloid onto carbon films (S160, 200 mesh Cu (25)) and allowing them to dry at room temperature.

UV-Vis extinction spectra were recorded using an Agilent 8453 single-beam diode array spectrometer. Measurements were performed for NS alone and NS bound to the intact biofilm surface where an untreated biofilm of the same age was used a blank sample.

**1.5 SERS analysis**

**1.5.1 *Ex-situ* biofilm SERS measurements**

*Ex-situ* biofilm measurements were conducted using an Avalon RamanStation R2 benchtop Raman spectrometer equipped with a 785 nm diode laser operating at 160 mW power (10 × 10 s accumulation time). For these experiments, 25 µL of the *ex-situ* biofilm was added to 175 µL of the NS colloid and the mixture allowed to react for 3 hours. Then, 25 µL of Levo solution was added, resulting in a final drug concentration of 10^-3^ mol dm^-3^.

**1.5.2 *In-situ* biofilm SERS measurements**

*In-situ* biofilm measurements were performed using a WiTec alpha300s Raman microscope with a 50 s accumulation time, a laser power of 5 mW (785 nm) and a 10x objective lens. For these experiments, 15 μL of a Levo solution (10⁻³ mol dm^-3^) was applied to the biofilm containing surface-bound NS, and SERS spectra were recorded at the specific spot where the Levo was applied. Similarly, the same volume and concentration of Levo solution were applied to the biofilm containing the embedded NS layer and SERS spectra were recorded at the application site every 5 minutes to monitor Levo penetration.

All spectra in this work were presented with minimal preprocessing, limited to intensity scaling for display purposes and averaging to ensure more reliable and consistent results.

**2. Figures**


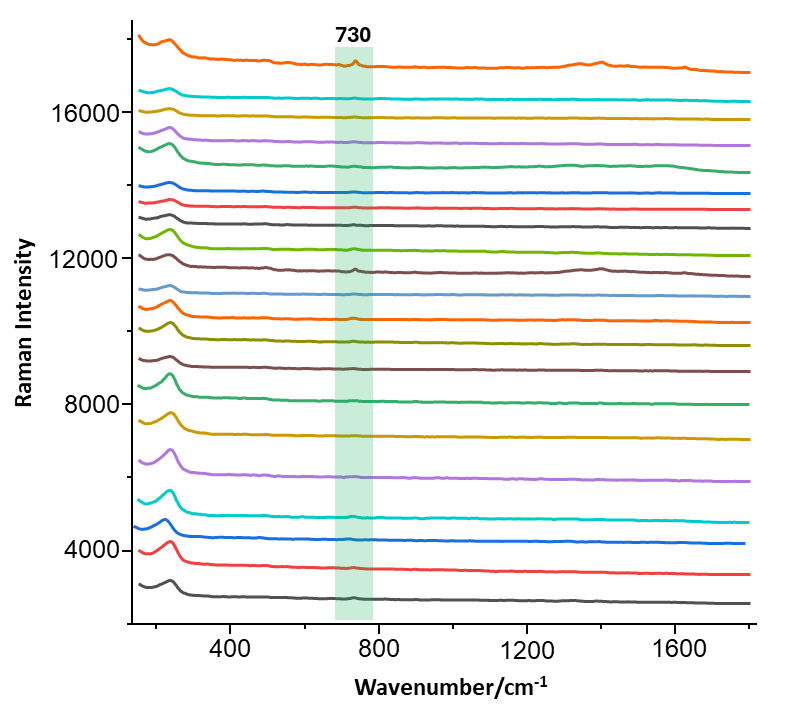


**Figure S1.** SERS spectra collected from different points on the surface of the intact biofilm treated with NS

*
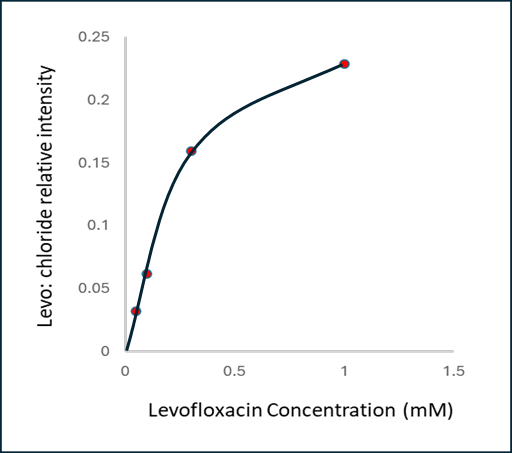
*

**Figure S2.** Plot of the relative intensity of the Levo band at 1395 cm^-1^ and chloride band at 236 cm^-1^ vs. Levo concentration established for the ex-situ biofilms.


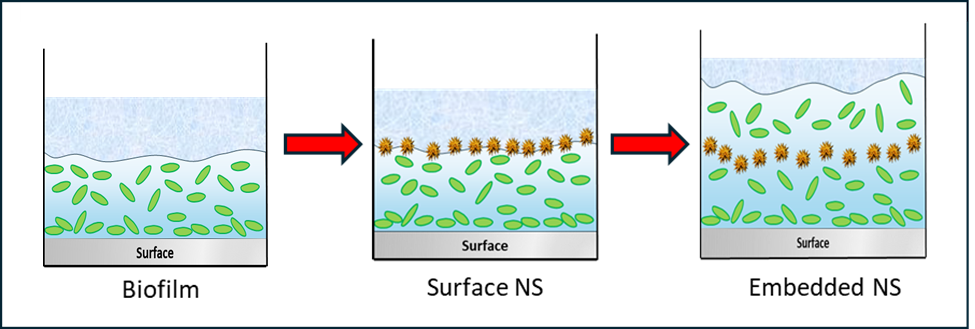


**Figure S3.** Schematic representation of the process for incorporating NS within the biofilm matrix.

**References**

[46] S. He, Y. M. E. Kyaw, E. K. M. Tan, L. Bekale, M. W. C. Kang, S. S. Y. Kim, I. Tan, K. P. Lam, J. C. Y. Kah, “Quantitative and Label-Free Detection of Protein Kinase A Activity Based on Surface-Enhanced Raman Spectroscopy with Gold Nanostars” *Anal. Chem.* 2018, 90 (10), 6071–6080.

[47] P. B. Flynn, W. G. Graham, B. F. Gilmore, “Acinetobacter Baumannii Biofilm Biomass Mediates Tolerance to Cold Plasma” *Letters in Applied Microbiology* 2019, 68(4), 344–349.
